# Nutcracker syndrome: a case-based review

**DOI:** 10.1308/rcsann.2023.0090

**Published:** 2023-12-01

**Authors:** D Maharaj, SR Mohammed, K Caesar, S Dindyal

**Affiliations:** ^1^ St Clair Medical Centre, Trinidad and Tobago; ^2^ University of the West Indies, Trinidad and Tobago; ^3^ Mid and South Essex NHS Foundation Trust, UK

**Keywords:** Nutcracker syndrome, Endovascular, Review

## Abstract

The nutcracker phenomenon, also known as left renal vein entrapment, occurs when there is extrinsic compression of the left renal vein, most often between the abdominal aorta and the superior mesenteric artery. Nutcracker syndrome refers to the constellation of clinical symptoms that may arise from the nutcracker phenomenon, typically inclusive of haematuria, flank/pelvic pain, orthostatic proteinuria and (in male patients) varicocele. We provide a short review of the nutcracker syndrome including various diagnostic and therapeutic modalities. We utilise our own experience with a patient as a case study and highlight the modern management option of endovascular stenting.

## Introduction

The earliest anatomical description of the nutcracker phenomenon was by Grant in 1937: “[…] the left renal vein, as it lies between the aorta and superior mesenteric artery, resembles a nut between the jaws of a nutcracker.”^1^ The terms ‘nutcracker phenomenon’ and ‘nutcracker syndrome’ were used interchangeably in the literature for many years^2^ but in recent years, emphasis has been placed on reserving the term ‘nutcracker syndrome’ for use only when there are clinical symptoms associated with the nutcracker phenomenon.^3–7^

The prevalence of nutcracker syndrome (NCS) globally is unknown as the lack of a consensus around diagnostic criteria means that it is likely underdiagnosed.^3^ NCS can occur in all ages but typically presents in the second to fourth decades of life. While it was once thought to be more common in women, recent data suggest that it affects both sexes relatively equally.^4,8^

## Case presentation

A 65-year-old woman presented with a history of moderate-to-severe pelvic pain and bilateral flank pain or the past several years. She had experienced deep dyspareunia and pelvic pain following intercourse for a similar timeframe. She described two bouts of macroscopic haematuria. There was no significant medical or gynaecological history.

**Figure 1:**
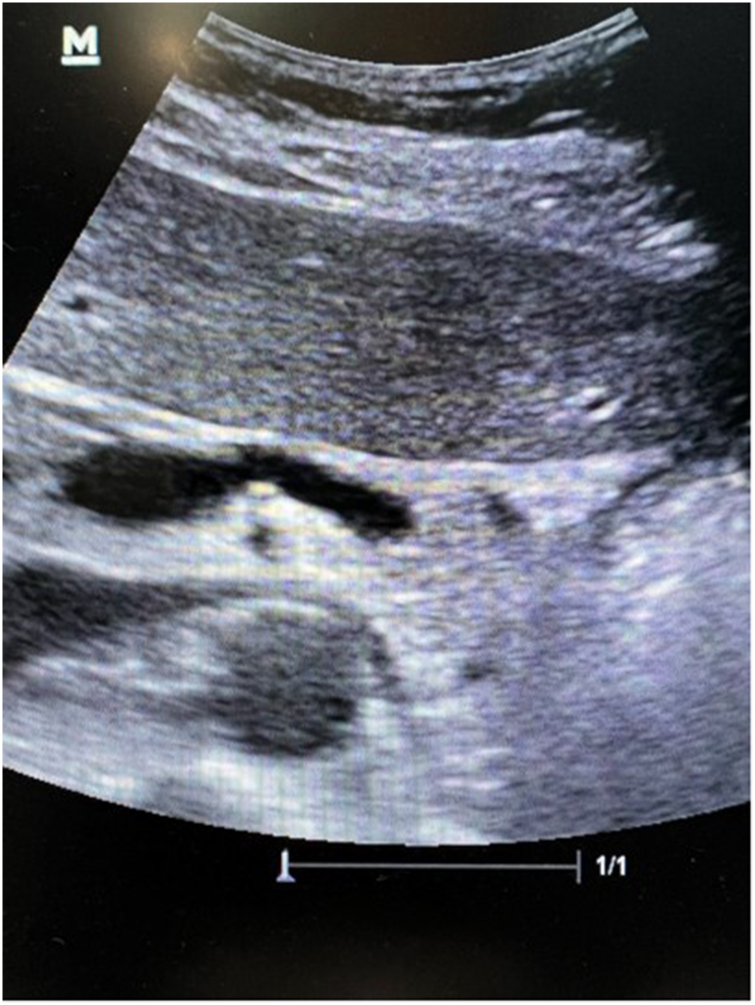
Duplex ultrasonography demonstrating narrowing of the left renal vein between the aorta and superior mesenteric artery

A renal function test revealed a creatinine level of 0.62mg/dl. Abdominopelvic ultrasonography showed prominent and dilated paraovarian vessels with narrowing of the left renal vein (LRV) between the aorta and superior mesenteric artery (SMA) (Figure 1). The patient underwent contrast-enhanced computed tomography, which additionally demonstrated a reduced angle between the aorta and SMA of approximately 25° with compression of the LRV and dilated bilateral ovarian veins with pelvic varices. Venography revealed no flow in the proximal LRV (Video 1 – available online, Venography displaying no flow in proximal left renal vein) with resultant escape retrograde flow down the left ovarian vein (Video 2 – available online, Venography demonstrating retrograde flow in left ovarian vein) and the pelvic veins before emptying into the inferior vena cava (IVC) via the right ovarian vein (Video 3 – available online, Venography demonstrating emptying into the inferior vena cava via the right ovarian vein). The LRV–IVC pressure gradient was elevated at 15mmHg.

The patient opted for immediate intervention and an endovascular approach was deemed suitable. The LRV was selected with a 5Fr Judkins right 3.5 catheter (Merit Medical, Salt Lake City, UT, US) over a 0.035” hydrophilic Glidewire^®^ (Terumo, Somerset, NJ, US). A 5Fr pigtail catheter was inserted into the contralateral groin, facilitating simultaneous venography of the IVC and LRV (6cm long, 12mm diameter). An Amplatz Extra-Stiff 0.035” wire (Cook Medical, Bloomington, IN, US) was advanced to the segmental renal veins and a 10Fr SafeSheath^®^ (Pressure Products, San Pedro, CA, US) parked in the IVC. A 14mm × 6cm venous Wallstent™ (Boston Scientific, Marlborough, MA, US) was deployed immediately proximal to the ostium of the left ovarian vein to just beyond the IVC (Video 4 – available online, Deployment of Wallstent™ proximal to ostium of the left ovarian vein). Venography confirmed resolution of the stenosis and reduced reflux into the left ovarian vein with no flow into the pelvic veins and right ovarian vein (Video 5 – available online, Venography confirming resolution of stenosis and reduced reflux into the left ovarian vein).

The patient experienced immediate cessation of flank and pelvic pain but noted onset of a vague, mild back pain, which lasted one week before dissipating. She was asymptomatic at a follow-up visit and venous duplex ultrasonography revealed a patent stent.

## How does left renal vein entrapment present clinically?

The nutcracker phenomenon, also known as LRV entrapment, occurs when there is extrinsic compression of the LRV, most often between the abdominal aorta and the SMA.^4,5^ NCS refers to the constellation of clinical symptoms that may arise from the nutcracker phenomenon, typically inclusive of haematuria, flank/pelvic pain, orthostatic proteinuria and (in male patients) varicocele.

NCS may be subdivided into two main variants: anterior NCS and posterior NCS. Anterior NCS (or classical NCS) refers to compression of the LRV between the abdominal aorta and SMA whereas posterior NCS (a far less common variant) occurs when the retro-aortic or circum-aortic renal vein is compressed between the aorta and vertebral body.^4,7,8^ Less common aetiologies of anterior NCS include pancreatic neoplasm, compression by adjacent lymphadenopathy, lordosis, and decreased retroperitoneal and mesenteric fat tissue.^5,7^

## How is nutcracker syndrome diagnosed?

There exist no consensus diagnostic criteria for NCS and it is therefore considered a diagnosis of exclusion. A history of haematuria and flank pain may precipitate urine studies such as urine analysis, urine phase contrast microscopy, urine culture and cytology. Doppler ultrasonography (DUS), computed tomography (CT) and magnetic resonance imaging (MRI) are all reasonable first-line radiological studies.^3–7^ Variations of normal anatomy may be visualised and should not immediately prompt a diagnosis of NCS. Retrograde venography/phlebography, with or without intravascular ultrasonography, is considered the gold standard for diagnosis, with an elevated pressure gradient between the LRV and inferior vena cava (IVC) confirming venous hypertension.

DUS is often recommended as the first-line study, with reported sensitivity and specificity ranging from 69% to 90% and from 89% to 100% respectively.^8,9^ Findings in DUS vary depending on patient positioning and technical difficulties (small sampling area, requirement that the patient be fasted for 6–8 hours and interobserver variability),^7,9^ limiting its usage.^8^ DUS enables real-time assessment of flow and peak velocities of the LRV. Various ratios of peak systolic velocity of the aortomesenteric segment to the hilar portion have been proposed as being significant/diagnostic, usually from 4.0 to 5.0.^7,9^

In instances where DUS is not diagnostic, axial imaging may provide greater accuracy. Cross-sectional imaging relies on both SMA angle and vessel diameter. The normal angle between the aorta and SMA (aortomesenteric angle) ranges between 40° and 90°, and varies with age and visceral fat area.^10,11^ Zhang *et al* reviewed 20 patients with NCS and suggested that an angle of <35° in the sagittal plane is significant for diagnosis of NCP.^12^ Kim *et al* studied 27 patients (12 with non-compensated NCS, 6 with partially compensated NCS and 9 controls).^13^ They reported that an aortomesenteric angle on sagittal CT at a cut-off of 41° had a sensitivity of 100% and a specificity of 55.6% for the diagnosis of NCS. The beak sign (abrupt narrowing of the LRV with an acute angle) showed 91.7% sensitivity and 88.9% specificity when differentiating non-compensated NCS from the control group.

Owing to absolute vessel diameter values varying for each individual, the ratio of the diameters of the LRV at the hilum and the narrowed aortomesenteric segment is used instead. A LRV diameter ratio (hilar-aortomesenteric) of ≥4.9 demonstrated the greatest diagnostic accuracy.^13^

MRI findings closely resemble those afforded by CT (i.e. dorsolateral torsion of the left kidney, abnormal SMA, abnormally high course and compression or pre-stenotic dilation of the LRV and gonadal vein varices). MRI has the benefit of absence of radiation, preferable in some populations (e.g. children). Er *et al* assessed the value of several MRI sequences in 40 children with NCS, and compared the sequences according to anatomical depiction, measurability and pulsation artefact; T2-TRUFI (True Fast Imaging with Steady-State Free Precession) provided the best imaging quality.^14^

The normal pressure gradient between the LRV and IVC is <1mmHg, with one study of 50 patients finding only 1 patient with a pressure gradient of >1mmHg.^15^ Nishimura *et al* proposed a pressure gradient ≥3mmHg as indicative of LRV hypertension^16^ while Takebayashi *et al* correlated ultrasonographic data, renocaval pressure gradients and presence of collateral veins on retrograde venography in 44 patients with haematuria of unknown origin, and subsequently defined pressure gradients as: normal (<1mmHg), borderline LRV hypertension (1–<3mmHg) and LRV hypertension (≥3mmHg).^17^ An elevated pressure gradient of ≥3mmHg is the standard reference point.^7,18^

## What is the pathophysiology that leads to nutcracker syndrome?

LRV hypertension leads to retention of venous blood, venous congestion and therefore increased resistance against arterial influx. This results in the formation and engorgement of periureteral and peripelvic varices.^7^ Rupture of these thin-walled varices produces microscopic or macroscopic haematuria.^7,8,15^ LRV hypertension further produces mild subclinical immune injuries to the vessel walls and a subsequent immune cascade, which exaggerates the release of angiotensin II and norepinephrine.^7,8,19^ This alteration from the normal physiological response leads to orthostatic proteinuria.^7,20^ Flank and abdominal pain are additional consequences of the inflammatory cascade.^19^ Left flank pain may also arise from left ureteral colic associated with passage of blood clots through the ureter.^3^

Scholbach proposed that “midline congestion syndrome” (i.e. pain and derangement of the organs in the midline of the body) may arise when NCS forces the left renal blood to bypass the site of compression via the abundant collateral veins (the veins of the midline organs).^21^ This may result in symptoms such as headache, back and abdominal pain, and gonadal vein syndrome.^4,21^ The left gonadal vein may become engorged in severe cases of NCS, and as it communicates directly with the ovarian venous plexus and then the uterine venous plexus, it may progress to pelvic congestion syndrome, presenting as pain, fullness or discomfort of the pelvis.^7^ Some authors have proposed that LRV hypertension is the usual cause of varicoceles^22,23^ although it may not be associated with differences in testicular parameters, or frequency of initial or reoperative surgery.^24^

## What are the management options for nutcracker syndrome?

Management of NCS is dependent on the severity and expected reversibility of symptoms while considering the age of the patient.^3–7^ Management options include observation, pharmacotherapy and surgery. Endovascular treatment is surging in popularity owing to its minimally invasive nature.^3–7^

Conservative management is recommended for cases with mild haematuria and tolerable symptoms, and in patients aged ≤18 years. Growing individuals may experience symptom resolution as intra-abdominal and fibrous tissue increases at the SMA origin, thus ‘freeing’ the LRV. Pharmacotherapy in the form of angiotensin-converting enzyme inhibitors for treatment of orthostatic proteinuria, with or without aspirin for maximisation of renal perfusion, is commonly utilised in the paediatric population.^7,8^ Persistent symptoms despite conservative measures (for up to 24 months in patients aged ≤18 years and up to 6 months in adults) are an indication for surgical intervention.

In 2009, Wang *et al* reported a single-centre experience of 23 patients with NCS.^25^ Sixteen patients with mild and tolerable symptoms were treated conservatively, with eight being prescribed low-dose acetylsalicylic acid. Clinical improvement occurred in eleven (68.75%) of these, with total relief in two cases and partial relief in nine. No relief occurred in 5 patients (31.25%) after a mean follow-up duration of 41.2 months. In 2019, Miró *et al* described their experience of NCS in the paediatric population.^26^ They encountered 21 patients, all of whom initially received conservative treatment. This approach achieved resolution of symptoms in 16 cases (76.2%), with the remaining 5 cases requiring a surgical approach.

Several open surgical approaches to management of NCS have been published, including (but not limited to) LRV transposition, patch venoplasty without LRV transposition, nephropexy, gonadal vein transposition and renal autotransplantation.^5^ LRV transposition is the most performed intervention.

Reed *et al* described 23 patients with radiological evidence of LRV compression.^27^ Twelve patients were managed expectantly while eleven underwent LRV transposition. Over a mean follow-up period of 39 months, symptoms of flank pain and haematuria improved in 8/10 and 7/7 patients respectively. Two patients underwent reintervention at other centres. Varicoceles recurred in 2/3 patients.

In their series of 37 patients treated for NCS, Erben *et al* performed open surgery on 36 of these.^28^ Distal transposition of the LRV into the IVC was performed in 31 cases with adjunctive great saphenous vein patch or cuff used to enlarge the LRV or decrease tissue tension between the LRV and IVC in several cases. A relatively high percentage (32%) of patients required reintervention within 24 months, most frequently LRV stenting.

Wang *et al*, in the aforementioned study, performed LRV transposition in 7 of 23 patients.^25^ Three of these had postoperative complications, including paralytic ileus in two patients. Haematuria resolved in all patients who underwent LRV transposition but pelvic pain persisted in one patient.

Renal autotransplantation is the preferred surgical modality at some centres as it may result in normalisation of renal venous circulation and obviates renal ptosis.^7,29^ Salehipour *et al* performed renal autotransplantation on four patients with NCS, two of whom had previously undergone LRV transposition.^29^ Patients were followed up from 4 to 24 months with complete resolution of symptoms in all cases. Renal autotransplantation entails much more extensive dissection and a longer period of renal ischaemia than LRV transposition,^27^ and is likely associated with a higher risk of complications.

Endovascular treatment of NCS is becoming a preferred modality owing to its minimally invasive nature.^3–7^ Chen *et al* reported a series of 61 patients who underwent endovascular stenting (EVS) of the LRV and reported that symptoms either improved or resolved in 59 of these within 6 months; symptoms recurred in 1 patient.^30^ There was a median follow-up duration of 66 months and there were no significant cases of re-stenosis. However, there were three cases of stent migration (1 each into the right atrium, IVC and hilar LRV).

Wu *et al* reported their experience of 75 patients who were followed up for a mean period of 55 months and described 5 cases of stent migration (1 each into the right ventricle, right atrium and left side of the LRV, and 2 into the IVC).^31^ They highlighted the importance of accurate measurement of the anatomical parameters prior to surgery and appropriate stent sizing.

Avgerinos *et al* performed a retrospective chart review of 18 patients with NCS who underwent LRV stenting.^32^ Five of these had prior LRV transposition that had failed within a mean of 7.0±4.9 months. At an average follow-up of 41.4±26.6 months, five patients had persistent symptoms, of whom three had previously undergone LRV transposition surgery. However, three patients underwent stent reintervention at 5.8, 16.8 and 51.7 months owing to symptom recurrence or stent re-stenosis, and there were no cases of stent migration.

Perioperative complications of endovascular stenting are rare, as is re-stenosis, with the major possible complication being stent migration. Some have recommended the use of intravascular ultrasonography to aid in preoperative planning and sizing of the LRV stent.^33^ Although short and medium-term outcomes of endovascular stenting are promising, there is a paucity of data on long-term follow-up, be it for either symptom recurrence or stent migration.

A notable disadvantage of EVS is that patients must be on anticoagulation and antiplatelet therapy for a short time thereafter. A recommended regimen is low molecular weight heparin for 3 days after EVS, 30 days of clopidogrel and ≥3 months of aspirin.^4,30^

## Conclusions

NCS is a rare and likely underdiagnosed entity. Diagnosis can be made via a multitude of radiological methods, including DUS, CT and MRI, although retrograde venography remains the gold standard confirmation. Conservative treatment options may be used in the initial management. Definitive surgical management may be performed via several methods; endovascular management is surging in popularity globally owing to its minimally invasive nature, and lower rates of intra and postoperative complications.

## Ethical approval

Owing to the present study being a retrospective case report, ethical approval was not required. Informed consent was obtained from the patient for all procedures conducted as part of clinical care/management.
